# Degradation Reduces Microbial Richness and Alters Microbial Functions in an Australian Peatland

**DOI:** 10.1007/s00248-022-02071-z

**Published:** 2022-07-22

**Authors:** Christina Birnbaum, Jennifer Wood, Erik Lilleskov, Louis James Lamit, James Shannon, Matthew Brewer, Samantha Grover

**Affiliations:** 1 Applied Chemistry and Environmental Science, School of Science, RMIT University Melbourne, Victoria, 3001 Australia; 2 School of Life and Environmental Sciences, Faculty of Science & Built Environment, Deakin University, 221 Burwood Highway, Burwood, VIC 3125 Australia; 3 School of Agriculture and Environmental Science, The University of Southern Queensland, Toowoomba, QLD 4350 Australia; 4 Physiology, Anatomy and Microbiology, La Trobe University, Science Drive, Bundoora, VIC 3086 Australia; 5 USDA Forest Service, Northern Research Station, 410 MacInnes Dr, Houghton, MI 49931 USA; 6 Department of Biology, Syracuse University, 107 College Place, Syracuse, NY 13244 USA; 7 Department of Environmental and Forest Biology, State University of New York College of Environmental Science and Forestry, 1 Forestry Drive, Syracuse, NY 13210 USA; 8 Research Centre for Applied Alpine Ecology, Department of Ecology, Environment and Evolution, La Trobe University, Bundoora, VIC 3086 Australia

**Keywords:** Peatland, Archaea, Bacteria, Mycorrhizae, Decomposition, Soil carbon

## Abstract

Peatland ecosystems cover only 3% of the world’s land area; however, they store one-third of the global soil carbon (C). Microbial communities are the main drivers of C decomposition in peatlands, yet we have limited knowledge of their structure and function. While the microbial communities in the Northern Hemisphere peatlands are well documented, we have limited understanding of microbial community composition and function in the Southern Hemisphere peatlands, especially in Australia. We investigated the vertical stratification of prokaryote and fungal communities from Wellington Plains peatland in the Australian Alps. Within the peatland complex, bog peat was sampled from the intact peatland and dried peat from the degraded peatland along a vertical soil depth gradient (i.e., acrotelm, mesotelm, and catotelm). We analyzed the prokaryote and fungal community structure, predicted functional profiles of prokaryotes using PICRUSt, and assigned soil fungal guilds using FUNGuild. We found that the structure and function of prokaryotes were vertically stratified in the intact bog. Soil carbon, manganese, nitrogen, lead, and sodium content best explained the prokaryote composition. Prokaryote richness was significantly higher in the intact bog acrotelm compared to degraded bog acrotelm. Fungal composition remained similar across the soil depth gradient; however, there was a considerable increase in saprotroph abundance and decrease in endophyte abundance along the vertical soil depth gradient. The abundance of saprotrophs and plant pathogens was two-fold higher in the degraded bog acrotelm. Soil manganese and nitrogen content, electrical conductivity, and water table level (cm) best explained the fungal composition. Our results demonstrate that both fungal and prokaryote communities are shaped by soil abiotic factors and that peatland degradation reduces microbial richness and alters microbial functions. Thus, current and future changes to the environmental conditions in these peatlands may lead to altered microbial community structures and associated functions which may have implications for broader ecosystem function changes in peatlands.

**Supplementary Information:**

The online version contains supplementary material available at 10.1007/s00248-022-02071-z.

## Introduction

Peatland ecosystems play a central role in global C cycling, while also releasing carbon dioxide (CO_2_) and methane (CH_4_) to the atmosphere via decomposition [1, 2]. Hydrology plays a critical role in balancing the accumulation or decomposition of plant material in peatlands. For example, in anoxic conditions peat soil can accumulate when water-logging hinders the breakdown of plant material resulting in plant growth exceeding decomposition [3, 4]. Climate change is affecting the C dynamics in both natural and disturbed peatlands as has been reported in a number of studies [5–7]. This has led to an increasing interest in peatland research and the above- and below-ground drivers of ecosystem processes which may facilitate restoring peatland ecosystem functions, including C sequestration, in disturbed peatlands [8].

Microbial communities and associated decomposition processes are vertically stratified with depth in peatlands, which is caused by changes in redox conditions, C quality, and oxygen (O_2_) availability [8–11]. The highest amount of decomposition, biological metabolism, and nutrient cycling occurs in the acrotelm (aerobic soil layer), followed by the mesotelm where conditions fluctuate between anoxic and oxic depending on the water table levels resulting in shifting metabolic processes [12–16]. Finally, litter enters the water-saturated catotelm, which is permanently anoxic, where biological material is decomposed slowly, predominantly by prokaryotes [17]. These changes in vertical abiotic conditions are likely to lead to changes in fungal and prokaryotic community composition, diversity, and function. Fungi are important organic matter decomposers in peatlands influencing carbon dynamics via the synthesis of extracellular enzymes [18]. The decomposer fungi, consisting of pathogens and weak parasites, pioneer saprobes, polymer degraders, recalcitrant polymer degraders, and secondary saprobes have been comprehensively described before [18]. However, our understanding of the mycorrhizal and free-living saprotrophic fungi, involved in peatland carbon cycling, is still very limited [18–20] in comparison with the prokaryotic guilds (e.g., methanogens and methanotrophs) that have received more research attention [21, 22].

Knowledge to date suggests that in the oxic upper layers, i.e., the acrotelm, aerobic decomposition is driven by saprotrophic fungi rather than bacteria [19–21]. As organic matter is degraded from litter to less labile organic matter, it promotes succession of fungal communities with different growth traits and enzymatic capabilities, e.g., from saprotrophic to mycorrhizal communities [22, 23]. Similarly, bacterial communities have been reported to change in composition and specialization along a vertical soil gradient with a general decrease in their diversity [8, 10, 11, 21, 24]. Specifically, at intermediate depths in peatlands, the mesotelm, which has been termed the “hot spot” of microbial diversity and activity, lies at the interface between oxic and anoxic layers where the water table fluctuates and the simultaneous presence of CH_4_ and O_2_ facilitates conditions for methanotrophic bacteria (methanotrophs) [8]. Methanotrophs metabolize CH_4_ to produce biomass and CO_2_ [25]. Taken together, microbial communities in peatlands play an important role in greenhouse gas emissions, especially CO_2_ and CH_4_, as well as the ongoing sequestration of carbon in the peat itself and thus it is important to understand belowground microbial diversity and ecology to better predict global C dynamics, especially in a warming climate.

The balance between greenhouse gas emissions and carbon sequestration in peatlands may shift as a result of disturbance (e.g., grazing, fire, changes in hydrology, nutrient deposition, increased temperatures). For example, drainage of peatlands for agricultural/grazing purposes has been a management strategy in many countries which leads to changes to water table regime [8]. Water table position fluctuations or declines in peatlands will be accompanied by subsequent changes to the microbial communities and their functioning [8, 26–28]. For instance, fungal communities have been shown to decline in richness and diversity following drainage and changes in water table depths [29, 30]. However, fungal communities play an important role after peatland drainage supporting the establishment of tree species, especially with the encroachment of forest.

Bacterial composition has been shown to be unaffected by short-term water table drawdown, whereas long-term changes to water table did cause shifts in bacterial composition [31, 32]. Drainage has been shown to decrease the abundance of methanotrophs whose ecological niche is dependent on the position of the water table [33]. Overall, previous studies suggest that drainage of peatlands may decrease the diversity and alter the net functioning of fungal and bacterial communities across the soil depth gradient (i.e., acrotelm, mesotelm, and catotelm).

The objective of this study was to compare prokaryotic and fungal community structure from two bogs located in the Australian Alps on Wellington Plains peatland (one degraded, here referred to as “dried” following historic grazing activity, the other intact) along a vertical soil depth gradient. Peat soils in the Australian Alps comprise a mosaic of intact bog peats and disturbed dried peats [34]. Dried peat in this region is likely to be derived from bog peat as a consequence of bog drainage caused by cattle grazing damage [35]. Peat soils have attracted renewed attention following bushfires (1998, 2003, 2006) and an end to cattle grazing (2005) in the Australian Alps which has led to peatland restoration work [36–38]. Understanding the belowground microbial structure and associated carbon cycling in peat soils is central to conducting informed rehabilitation works in the Australian Alps [39].

Therefore, we assessed fungal and prokaryotic community structure and predicted prokaryotic functional gene types using PICRUSt and fungal guilds using FUNGuild. We hypothesized that prokaryotic and fungal structure will (1) be vertically stratified in the intact bog and (2) differ in degraded dried peat as compared to the intact bog peat due to the variation in the environmental and edaphic factors. The microbial communities in Australian peatlands are extremely understudied and we lack a baseline knowledge on the microbial diversity in these soils. Thus, our results will contribute to better understanding the effect of disturbance on belowground microbial dynamics in peatlands and the associated effects on C turnover and inform scientifically grounded management and restoration of peatlands.

## Methods

### Study Site Description and Sample Collection

**Fig. 1:**
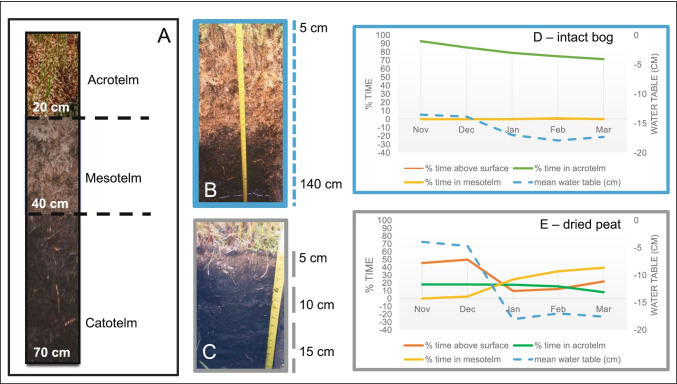
Schematic representation of acrotelm, mesotelm, and catotelm peat profile and soil depths (a). Profile photo of intact bog (b) and dried peat (c) from [40] showing peat profiles from the Wellington Plains peatland adjacent to our sampling sites. Reproduced with permission. Mean water table position (cm) and mean % time the water table is at different depths from November to March (plant growing season) in the intact bog (d) and dried peat (e) sites in Wellington Plains peatland, Australia, is shown for the period 2008–2015. In intact bog (d), the water table is 70–90% of time in the acrotelm and never above the surface, whereas in the dried peat the water table residency % time fluctuates throughout the year and the water table level is 10–50% of time above surface, especially in November and December (E). Peat sampling in dried peat and intact bog was conducted in March 2015

Our study site is located in the Australian Alps on Wellington Plains peatlands. Detailed description of Wellington Plains location (see Fig. 1. in [40]), geomorphology, and vegetation can be found in [39]and [1]. Briefly, the peatlands occur beneath springs and seepages where groundwater reaches the surface and also along the valley bottom [35]. The carbon chemistry and decomposition processes of these peats are described in [1, 39]. The physical and chemical properties of the peat from Wellington Plains are comparable with those of *Sphagnum* peatlands in the northern hemisphere [41].

The vegetation composition of the peatland has remained stable since its formation [42]. The most extensive peat types are bog peat and dried peat; the dominant vegetation in bog peat is *Sphagnum cristatum*, *Empodisma minus*, *Baloskion australe*, *Epacris paludosa*, *Richea continentis*, *Baeckea gunniana*, *Astelia alpina*, and *Carex gaudichaudiana* and in the dried peat is *Empodisma minus*, *Baloskion australe*, *Poa costiniana*, *Epacris paludosa*, *Baeckea gunniana*, and *Carex gaudichaudiana* [35]. While there is much overlap in the vegetation between the two peat types, the main distinction between bog and dried peat vegetation is the lack of hydrophilic *Sphagnum* in the dried peat and the lack of *P. costiniana* in the bog peat [35]. Another important consideration is that due to the historic cattle grazing and drainage causing compaction and weathering, the dried peat acrotelm is narrower or absent compared to pristine intact bog acrotelm that has been undisturbed for millennia. Catotelm is often absent in the dried peat.

A total of twelve soil samples from intact bogs (− 37° 29′ 43″ N, 146° 49′ 44″ E) and degraded peatlands (− 37° 29′ 43″ N, 146° 49′ 52″ E) were sampled on 7–8 March 2015. Our samples are a subset of those from the Global Peatland Microbiome Project, a larger study aimed at characterizing microbial communities and biogeochemistry in the Earth’s peatlands (Lamit and Lilleskov et al. unpublished, [43]). Intact bog samples were collected from triplicate sites at three depths: 10–20 cm (acrotelm), 30–40 cm (mesotelm), and 60–70 cm (catotelm) below the peat surface from pits excavated by hand using serrated knife [43]. In the dried peat, samples were collected from triplicate sites at 10–20 cm only, due to shallow peat profiles. Soil sampling equipment was carefully sterilized with ethanol between replicates or replaced between each sample collection and between site. Peat samples were kept on dry ice in a cooler in the field before being transferred to storage at − 80 °C upon returning to the laboratory at La Trobe University the next day.

Mean water table position across years 2008–2015 in both dried peat and intact bog sites remained, on average, below the surface (between zero to − 15 cm) (Fig. 1 d and e). However, in the dried peat, the water table was above the surface for significantly longer than in the intact bog (dried peat: max = 5.3 cm, ± S. E = 0.2, intact bog: max =  − 2.7, ± S. E = 0.1) (Fig. 1 d and e). Notably, in the intact bog, the water table is on average 75–90% of time in the acrotelm and never above the surface, whereas in the dried peat the water table is 10–40% of the time above the surface (Fig. 1 d and e). Thus, the water table is more variable in the dried peat site. Variable water table can recharge alternate electron acceptors, driving more microbial degradation of peat under anoxic conditions, and altering microbial communities.

### Environmental and Soil Chemistry Data

Environmental data collected for each site and sample included *Sphagnum* and vegetation cover, pore water pH, electrical conductivity, and depth to water table (cm). Understory plant data was collected in 1m^2^ plots and canopy cover was taken above the core location (Table 1) [43]. Peat soil samples were analyzed for peat pH (2:1 water to peat slurry (volume to volume)) and a number of chemistry variables including C (%), N (%), S (%), Al (mg/kg), Ca (mg/kg), Fe (mg/kg), K (mg/kg), Mg (mg/kg), Mn (mg/kg), Na (mg/kg), P (mg/kg), Pb (mg/kg), Zn (mg/kg).

**Table 1:** Peatland environmental conditions and vegetation composition. pH, electrical conductivity, depth to water table (cm), total species richness, vegetation composition, and cover % are at a Core level. The mycorrhizal associations of listed species are in Supplementary Table 1

Core #	Peatland condition	Soil depth analyzed	pH	Electrical conductivity (µS/cm)	Depth to water table (cm)	Total plant species richness	Vegetation composition and cover %
Core 1	Intact	10, 30, and 60 cm	4.67	54	50	5	*• Baloskion australe* (40%), *Empodisma minus* (30%), *Epacris paludosa* (20%), *Baeckea gunniana* (10%), *Sphagnum cristatum* (100%)
Core 2	Intact	10, 30, and 60 cm	4.87	55	52	7	*• Empodisma minus* (45%), *Baloskion australe* (30%), *Epacris paludosa* (30%), *Baeckea gunniana* (10%) and *Astelia alpina* (2%), *Sphagnum cristatum* (80%), unidentified moss (5%)
Core 3	Intact	10, 30, and 60 cm	5.02	37	17	8	*• Empodisma minus* (60%), *Baeckea gunniana* (25%), *Baloskion australe* (20%), *Epacris paludosa* (20%), Unknown shrub sp. (15%), unidentified sp. (30%), *Sphagnum cristatum* (30%), unidentified moss sp. (70%)
Core 4	Dried	10 cm	4.35	NA	33	10	*• Empodisma minus* (40%), *Poa sp*. (30%), *Baloskion australe* (20%), *Baeckea gunniana* (10%), *Baeckea utilis* (10%), *Celmisia costiniana* (10%), *Hydrocotyle sp*., *Solenogyne dominii*, unidentified sp. (5%)
Core 5	Dried	10 cm	5.27	42	21	7	*• Empodisma minus* (50%), *Baloskion australe* (40%), *Epacris celata* (20%), *Celmisia costiniana* (5%), *Poa sp*. (30%), *Viola betonicifolia* (5%), unidentified moss sp. (30%)

Freeze dried soil samples were ground using a Wiley Mini Mill (Thomas Scientific, Swedesboro, NJ, USA) with stainless steel sieve (size 40 mesh) at Laurentian University (Sudbury, Ontario, Canada). One subsample from each peat sample was weighed (ca. 75 mg each on a balance with 0.01 mg readability) and pressed with W-oxide catalyst (ca. 150 mg) in Al foil prior to analysis on a VarioMacro CNS Analyzer (Elementar Gmbh, Langenselbold, Germany). Precision was confirmed using blanks and sulfadiazine for C, N, and S, and calibration was done with QA standards (NIST-1515-SRM).

A second ca. 1 g sub-sample was weighed in an acid-washed and “pre-ashed” ceramic crucible and combusted in a muffle furnace at 550 °C for 6 h. Remaining ash was weighed to determine loss on ignition and then digested two times at 110 °C for 3.5 h in 10 ml of HF and HCl (until dried), subsequently in HNO_3_ and HCl, and then in a mix of all 3 acids (each subsequent digestion step also lasted 3.5 h, allowing drying). Samples were eluted in 50 ml distilled deionized H_2_O and analyzed on a Varian 810 ICP-MS (Agilent Technologies, Santa Clara, CA, USA) for Al, Ca, Fe, K, Mg, Mn, Na, P, Pb, and Zn with dilutions to match to the instrument detection limits. Duplicate samples, blanks that were subjected to all processing steps minus peat samples, and standard organic reference material (NIST spinach and tomato leaves) were included every 20 samples to ensure data quality.

The water table was monitored in the bog peat and the dried peat using 2 TruTrack water height loggers (WT-HR; TruTrack Ltd, Christchurch, New Zealand) from 2008 to 2015. The TruTrack water height loggers were suspended in perforated 4 cm PVC tubing and logger position relative to the surface was recorded. The tubing was fitted with a cap to prevent rain or snow water from entering. The bog peat is deeper than the dried peat and thus a 100-cm-long WT-HR 1000 logger was used in the bog peat and a 25-cm-long WT-HR 250 was used in the dried peat. Both loggers were configured to read at 2 hourly intervals, and data transfer was carried out 6–9 monthly using Omni7 software (Trutrack Ltd, Christchurch, New Zealand). Note that Fig. 1 presents average water table data from TruTrack water loggers, whereas the water table data shown in Table 1 was measured at the time of soil sampling in 2015.

### Molecular Analysis and Bioinformatics

Methods for microbial DNA analyses are published in detail elsewhere [43, 44], so are described in brief here. DNA from peat samples was extracted using PowerSoil® DNA Isolation kit (Qiagen, USA) and PowerClean® kit (Qiagen, USA) following [43] and [44]. Amplicon library preparation and sequencing were conducted by The Department of Energy's Joint Genome Institute's protocol for Illumina MiSeq community amplicon sequencing (iTag; e.g., Tremblay et al. [45], protocol described in detail here: https://jgi.doe.gov/user-programs/pmo-overview/protocols-sample-preparaton-information/). Prokaryote (bacteria and archaea) and fungal communities were amplified using the V4 region of SSU rRNA with 515 (GTGCCAGCMGCCGCGGTAA) and 806 (GGACTACHVGGGTWTCTAAT) primer pairing [46] and ITS2 region with ITS9 (GAACGCAGCRAAIIGYGA) [47] and ITS4 (TCCTCCGCTTATTGATATGC) [48] primer pairing, respectively. 

Bioinformatic analyses were performed using QIIME2 [49]. Prior to denoising, primers were removed using Cutadapt and forward and reverse reads were truncated based on quality scores to 200 and 180 bp respectively for 16S data, and 240 and 200 bp respectively for ITS data. Denoising was performed using the QIIME2 DADA2 plugin which implements joining, quality filtering, and chimera detection [49, 50]. During denoising of ITS data, the 5.8S (94 bases) and 28S (35 bases) flanks were trimmed from the ITS reads. All amplicon sequence variants (ASVs) were aligned with mafft and used to construct a phylogeny with fasttree2 [51]. Taxonomic assignment for 16S and ITS ASVs was performed using the QIIME2 feature classifier plugin classify‐sklearn naïve Bayes classifier [52] trained on either the V4 regions of the 16S rRNA gene extracted from the Greengenes 13_8 99% OTU dataset [53] or on the full ITS region from the UNITE (v. 8.0, released 02.02.2019) dynamically classified reference dataset [54]. ITS data were further filtered by aligning ASV representative sequences against the UNITE reference data set using BLAST in QIIME2, and reads that did not match at least ≥ 70% of their length to fungi with a similarity of ≥ 75% were removed.

Prokaryote metabolic potential was based on abundances of MetaCyc metabolic pathways, predicted using PICRUSt2 [55]. Prokayotic metabolic pathway abundances were log10 transformed prior to downstream analyses. Fungi were assigned functional categories following ecological guild assignment using FUNGuild database [56]. After guild assignment, only ASVs that were assigned as “Highly Probable” and “Probable” were retained [57].

### Statistical Analyses

Prokaryotic and fungal composition, richness, and diversity were compared separately across the intact bog depth gradient (10, 30, and 60 cm, *n* = 9) and between intact and dried peat samples from 10 cm only (*n* = 5). Prior to analyses both ASV matrices were subsampled to equal depth using “rarefy_even_depth” without replacement function in “phyloseq” package (fungi to 72,558 reads and bacteria 146,570 reads per sample) [58]. To visually inspect whether soil depth and soil condition (intact vs dried peat) affected prokaryotic and fungal composition, non-metric dimensional scaling (NMDS) based on Bray–Curtis dissimilarity index was carried out on subsampled data generated from the prokaryotic and fungal species matrix. To quantify differences in prokaryotic and fungal composition based on ASV matrices, permutational multivariate analysis of variance (Permanova) was used with 9999 permutations in R package “vegan” with core set as a random factor [59]. Richness and diversity indices (i.e., observed ASV number, Chao1 index, Shannon index, and Inverted Simpson index) were summarized using “estimate_richness” function in “phyloseq” package [58] and square-root transformed prior to analyses if they did not meet the assumptions of data homogeneity. Indicator species were determined using the “multiplatt” function in “indicspecies” package with alpa value set to 0.05 and 999 permutations [60]. To assess the differences among soil depths and cores for the dependent variables, linear mixed models including cores, ASV richness, and diversity indices were built using “lmerTest” package [61]. For intact peatland site analysis, the permanova mixed models and linear mixed models included Depth as a fixed factor and Core as a random factor. Fixed effects were tested with Satterthwaite approximation. For intact vs dried peatland analysis, peatland condition was the fixed factor in permanova mixed model. One-way ANOVA was used to analyze richness and diversity of fungi and prokaryotes in the acrotelm of intact and dried peat samples.

To assess the effect of soil chemistry, sample depth relative to water table as a variable, and vegetation on prokaryotic and fungal community composition, we performed dbRDA (distance-based canonical redundancy analysis) using R package “vegan” [59] using the same fungal and bacterial dissimilarity matrices as for the NMDS. For all environmental variables, we first performed a forward selection using “ordistep” function with 9999 permutations to identify the environmental variables significantly explaining variation in community composition. Where dbRDA models were significant (Table 2), summaries of redundancy analyses (dbRDA) statistics for redundancy axes are presented. Additionally, a summary of permutation tests for dbRDA under the reduced model with 999 permutations for abiotic variables tested in the dbRDA analysis are presented. A heatmap and dendrograms for predicted prokaryote metabolic pathways were built with base R using the heatmap() function and scale set to “row” (i.e., soil depth). Water table position and log-transformed water table residency data was analyzed using one-way ANOVA. All plots were prepared using “ggplot2” [62] and “RColorBrewer” [63] packages and analyses were performed in R [64].

**Table 2:** Summary results for soil fungal and prokaryote structure in intact bog across three soil depths (10 cm, 30 cm, 60 cm) and between intact bog and dried peat acrotelm only (10 cm) from Wellington Plains, Australia. Composition and soil chemistry hydrology factors was analyzed using permanova mixed model and dbRDA, respectively

Response variable	Intact peatland		Intact vs dried peatland	
	*Fungi*	*Prokaryotes*	*Fungi*	*Prokaryotes*
	Depth *F* (df), *P*	Depth *F* (df), *P*	*F* (df), *P*	*F* (df), *P*
Composition	0.96 (2.8), 0.17	2.00 (2.8), **0.027**	1.92 (1.4), 0.2	3,04 (1.4), 0.1
Richness				
Observed ASV	0.001 (2.4), 0.99	2.08 (2.6), 0.21	0.6 (1.3), 0.49	12.03 (1.3), **0.04**
Chao1	0.02 (2.4), 0.98	2.08 (2.6), 0.20	0.54 (1.3), 0.51	12.05 (1.3), **0.04**
Diversity				
Shannon index	0.89 (2.6), 0.45	1.63 (2.6), 0.27	0.27 (1.3), 0.65	3.08 (1.3), 0.18
Inverted Simpson index	0.86 (2.4), 0.49	0.84 (2.6), 0.47	0.11 (1.3), 0.76	1.06 (1.3), 0.38
Soil chemistry and hydrology factors	1.58 (4.4), **0.001**	1.49 (5.3), **0.006**	1.24 (1.3), **0.03**	2.19 (3.1), 0.08

In bold are values that are significant at *P *< 0.05

## Results

### Soil Fungal and Prokaryote Community Structure and Diversity

**Fig. 2:**
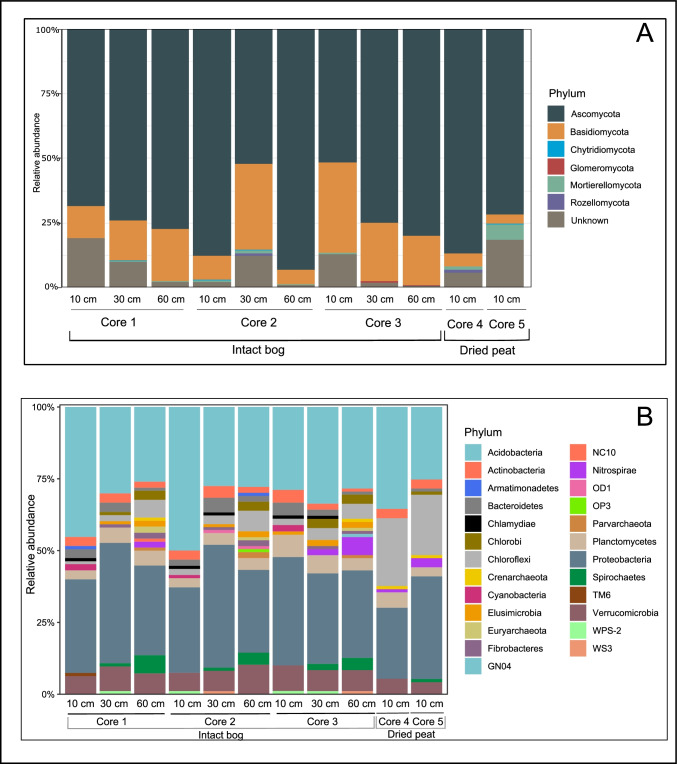
Relative abundance of fungi (a) and prokaryotes (b) (phylum level) in acrotelm (10 cm), mesotelm (30 cm), and catotelm (60 cm) from intact bog and dried peat cores from Wellington Plains peatland, Australia. Full list of fungal taxonomic assignments is available in Supplementary file 3. Relative abundances of dominant prokaryote phyla are available in Supplementary file 4

Fungal sequences were clustered into 1181 ASVs, which were further classified into six phyla with relative abundance > 0.01%. In the intact bog, members of the Ascomycota (73%) and Basidiomycota (19%) were the most abundant phyla on average across cores (Fig. 2a). Notably, the relative abundance of Basidiomycota was highest in the mesotelm and acrotelm from core 2 and core 3, respectively (Fig. 2a). In comparison, the relative abundance of Basidiomycota was lowest (4%) in the acrotelm in dried peat cores (Fig. 2a).

Prokaryotic sequences were clustered into 10,365 ASVs, which were further classified into 25 phyla with relative abundance of at least 1%. In the intact bog, members of Proteobacteria (31.6%) and Acidobacteria (30.8%) were the most abundant identified phyla on average across all sites, followed by Verrucomicrobia (6.8%) and Chloroflexi (6.7%) (Fig. 2b). Notably, the relative abundance of Chloroflexi was on average fivefold higher in dried peat (21%) than in intact bog cores (3.6%) (Fig. 2b).

**Fig. 3:**
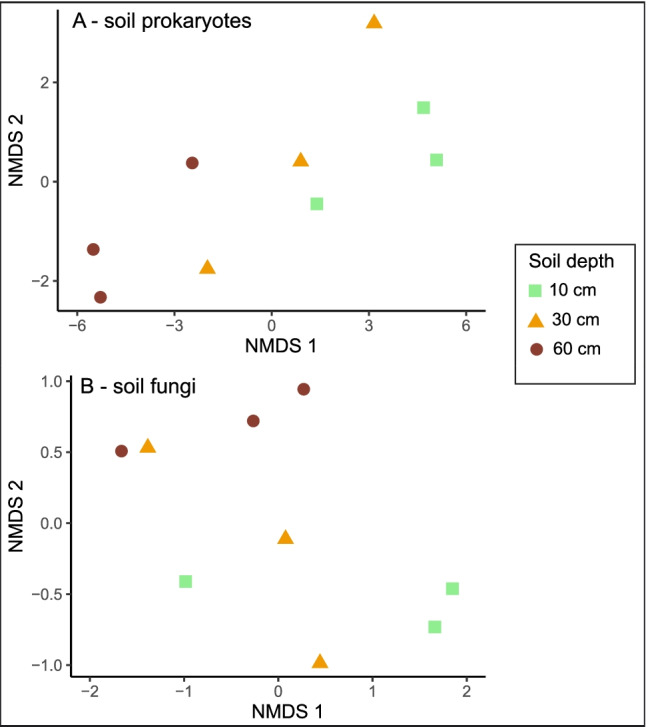
Non-metric multidimensional scaling ordination for (a) prokaryote and (b) fungal communities based on extracted DNA from intact bog soils collected from the acrotelm (10 cm), mesotelm (30 cm), and the catotelm (60 cm) from Wellington Plains peatland, Australia

We found that fungal community composition and richness in the intact bog were similar across the soil depth gradient, whereas soil depth had a significant effect on prokaryote community composition, with NMDS ordination revealing a significant clustering of prokaryotes by soil depth (Table 2 and Fig. 3 a and b). Prokaryote richness was significantly greater in the intact bog than in the dried peat (Table 2).

Fungal and prokaryote alpha diversity was not significantly influenced by the soil depth gradient in the intact bog (Table 3). However, we did find that prokaryote richness was significantly higher in the intact bog compared to dried peat (Table 3).

**Table 3:** Alpha diversity indices (mean ± SE) at different peat depths from intact (*n* = 3) and dried peat (*n* = 2) cores at Wellington Plains peatland, Australia. Different letters indicate significant difference (*P* < 0.05) between intact bog and dried peat prokaryote richness in the acrotelm

Depth (cm)	Peatland condition	ASV richness	Chao 1 index	Shannon’s diversity	Inverted Simpson’s index
Prokaryotes					
10–20	Intact	1599 ± 101^**a**^	1600 ± 101^**a**^	6 ± 0	220 ± 37
30–40	Intact	1916 ± 64	1917 ± 65	7 ± 0	295 ± 16
60–70	Intact	1537 ± 212	1538 ± 212	6 ± 0	229 ± 66
10–20	Dried	1044 ± 124^**b**^	1044 ± 124^**b**^	6 ± 0	164 ± 36
Fungi					
10–20	Intact	178 ± 17	183 ± 15	3 ± 0.3	11 ± 3
30–40	Intact	180 ± 27	182 ± 28	3 ± 0	11 ± 1
60–70	Intact	179 ± 25	180 ± 25	3 ± 0	8 ± 0
10–20	Dried	212 ± 51	216 ± 54	3 ± 1	9 ± 7

Indicator species analysis revealed that a total of 72, 10, and 102 prokaryote species associated significantly with the acrotelm, mesotelm, and catotelm in the intact bog, respectively. For fungi in the intact bog, indicator species analysis revealed that *Taphrina carpini* (Taphrinaceae) and *Symmetrospora gracilis* (Symmetrosporaceae) associated significantly with the catotelm. *Trichoderma sp.* (Hypocreaceae) associated significantly with both acro- and mesotelm, whereas *Hypochnicium huinayensis* (Podoscyphaceae) associated significantly with meso- and catotelm.

### Soil Fungal and Prokaryote Functional Diversity

**Fig. 4:**
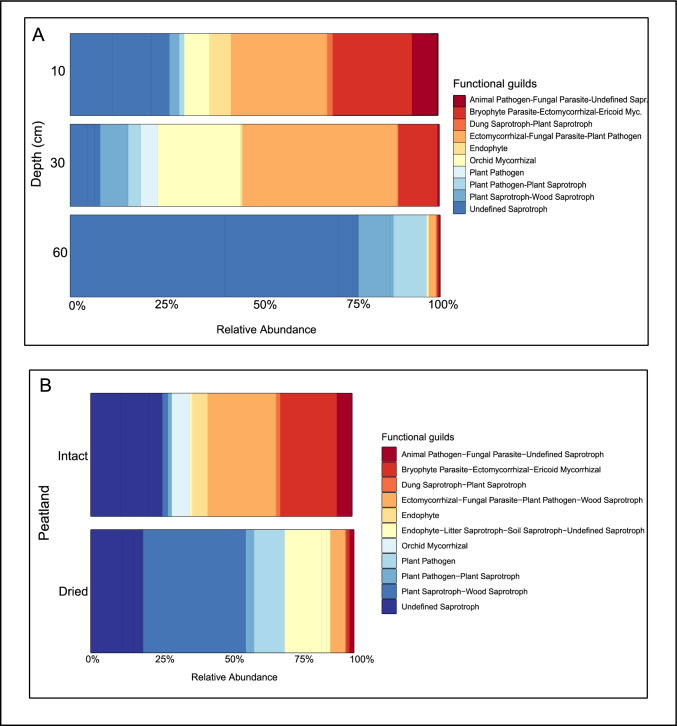
Guild level relative abundance of the soil fungal community in the acrotelm, mesotelm, and catotelm in the intact bog (a) and in the acrotelm of the intact bog and dried peat (b) from Wellington Plains peatland, Australia

Fungal functional guilds showed shifts among soil depths in the intact bog (Fig. 4a). The relative abundance of plant pathogens and saprotrophs was notably highest in the catotelm (60 cm) and lowest in the mesotelm (30 cm). Conversely, the relative abundance of mycorrhizas was lowest in the catotelm and highest in the mesotelm (Fig. 4a).

The differences in the relative abundance of soil fungal functional guilds were apparent between the intact bog and dried peat (Fig. 4b). Specifically, the relative abundance of plant pathogens/saprotrophs was two-fold higher in the dried peat acrotelm compared with the intact bog acrotelm (Fig. 4b).

**Fig. 5:**
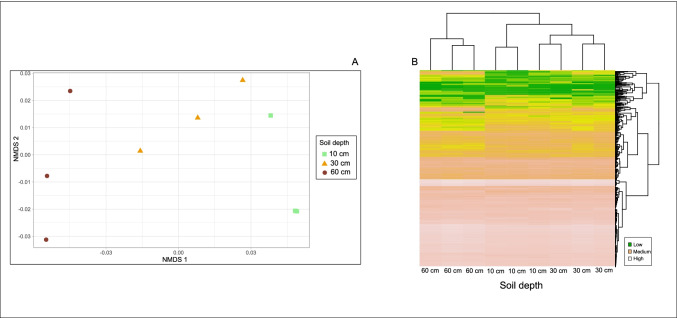
Non-metric multidimensional scaling ordination (a) and heatmap (b) for predicted bacterial 420 KEGG pathways based on extracted DNA from intact bog soils from the acrotelm, mesotelm, and catotelm from Wellington Plains peatland, Australia. The heatmap with dendrograms is scaled by “row” which corresponds to predicted metabolic pathway. The column corresponds to soil depth. Each observation is a row and each square is a value. For full list of predicted metabolic pathways shown in panel B, please see the Supplementary file 2

PiCRUSt generation of Kyoto Encyclopedia of Genes and Genomes (KEGG) assigned predicted functional content (i.e., predicted metagenome content) to 420 KEGG pathways based on the Illumina sequencing reads. Metabolic pathways were significantly different among the acro-, meso-, and catotelm in the intact bog (F_2,8_ = 6.5, *P* = 0.03, Fig. 5a), but not between the acrotelms of the intact bog and the dried peat (F_2,4_ = 7.36, *P* = 0.1). The heatmap dendrogram revealed that the catotelm metabolic profile differed from the acro- and mesotelm metabolic profiles (Fig. 5b), which were more similar and grouped together.

Indicator species analysis revealed that ten prokaryote predicted metabolic pathways contributed significantly to metabolic divergence in samples from the mesotelm and catotelm from intact bog sites (Table 4). Notably, these ten pathways were, on average, fivefold more abundant in the catotelm than in the mesotelm (Table 4). Nucleoside and nucleotide degradation (PWY-5532) and 7-(3-amino-3-carboxypropyl)-wyosine biosynthesis (PWY-7286) pathways were absent from all acrotelm soils, while sulfoquinovose degradation I (PWY-7446), L-rhamnose degradation II (PWY-6713), chorismate biosynthesis II (PWY-6165), tetrahydromethanopterin biosynthesis (PWY-6148), L-tryptophan biosynthesis (PWY-6629), lipopolysaccharide biosynthesis (LPSSYN-PWY) and METHANOGENESIS-PWY were absent in two out of three acrotelm soil samples from intact bog sites. Note that at least four of these pathways upregulated in the catotelm are associated with archaeal metabolism, at least two of which (PWY-6148, METHANOGENESIS-PWY) are associated with methanogenesis.

**Table 4:** Ten bacterial predicted metabolic pathways that contributed significantly to soils from the mesotelm and catotelm from intact bog sites, Wellington Plains peatland, Australia. Predicted metabolic pathway description is based on the MetaCyc searches (metacyc.org)

Predicted metabolic pathway name	Predicted metabolic pathway description	Indicator value	*p*—value	Relative average abundance in mesotelm	Relative average abundance in catotelm
PWY-5532	Nucleoside and nucleotide degradation (archaeal)	1.00	0.03	3.1%	28.3%
PWY-7286	7-(3-amino-3-carboxypropyl)-wyosine biosynthesis	1.00	0.03	2.2%	29.4%
PWY-7446	Sulfoquinovose degradation I	0.98	0.03	6.8%	26.2%
PWY-6713	L-rhamnose degradation II	0.98	0.03	7.9%	24.9%
PWY-6165	Chorismate biosynthesis II (archaeal)	0.97	0.03	3.9%	29.3%
PWY-6148	Tetrahydromethanopterin biosynthesis, co-Enzyme in methanogenesis by archaea	0.97	0.03	2.3%	30.9%
PWY-6629	L-tryptophan biosynthesis	0.96	0.03	6.3%	26.8%
LPSSYN-PWY	Lipopolysaccharide biosynthesis	0.96	0.03	7.5%	25.5%
METHANOGENESIS-PWY	Methanogenesis from H_2_ and CO_2_ (archaeal)	0.96	0.03	4.9%	26.9%
METHGLYUT-PWY	Methylglyoxal degradation	0.95	0.02	6.6%	25.9%

### Abiotic and Biotic Controls on Soil Fungal and Prokaryote Composition

**Fig. 6:**
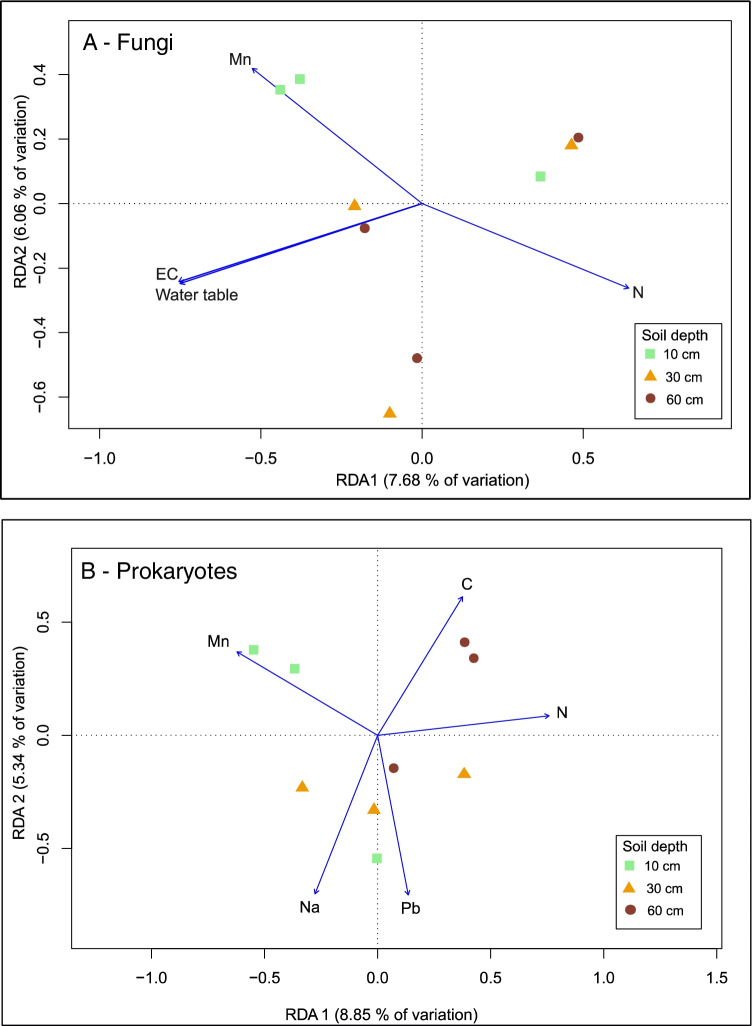
dbRDA biplot for fungal (a) and prokaryote (b) communities in intact bog soil from acrotelm, mesotelm, and catotelm based on extracted DNA from soil from Wellington Plains peatland, Australia

Both fungal and prokaryotic composition was significantly driven by soil chemistry and hydrology factors in the intact bog as revealed by dbRDA analysis (Table 2). Specifically, the fungal dbRDA model explained 22.5% (*R*^2^_adj_) of the overall variation in the fungal composition, with dbRDA axes 1 and 2 accounting for 7.68% and 6.06% of this effect, respectively (Table 2, Fig. 6a). RDA axis 1 was statistically significant, whereas RDA axis 2 was near significant (Supplementary Table 5). Manganese (Mn), nitrogen (N), electrical conductivity (EC) and water table level (cm) explained best the fungal variation along the RDA axis 1 (Fig. 6a). The permutation test for the soil chemistry and hydrology factors tested in the dbRDA analysis revealed that Mn, EC and water table level (cm) were statistically significant (Supplementary Table 6). For example, we found that mean Mn (mg/kg) concentration was at least tenfold higher in the acrotelm 331. 9 (± 162.2) (mg/kg), compared with the mesotelm 39.9 (± 15.1) mg/kg and catotelm 15.5 (± 6.8) mg/kg (Supplementary Fig. 2).

The prokaryote dbRDA was statistically significant for prokaryote composition in the intact bog sites (Table 2, Fig. 6b). This model explained 23.57% (*R*^2^_adj_) of the overall variation in the prokaryote composition, with dbRDA axes 1 and 2 accounting for 8.85% and 5.35% of this effect, respectively. RDA axis 1 was statistically significant (F_1,8_ = 2.80, *P* = 0.009), whereas RDA axis 2 was not significant (F_1,8_ = 1.69, *P* = 0.22, Supplementary Table 7). Peat carbon (C), nitrogen (N), lead (Pb), sodium (Na), and manganese (Mn) best explained the bacterial variation along the RDA axis 1 (Fig. 6b.). However, the permutation test for the five soil chemistry factors tested in the dbRDA analysis revealed that these factors were not significant, suggesting that a combination of these factors rather than any single tested factor best explains the overall effects on bacterial composition across the three soil depths (Supplementary Table 8).

The fungal dbRDA model for intact and dried peat samples explained 5.6% (*R*^2^_adj_) of the variation in the fungal composition (Table 2, Supplementary Fig. 1). Iron (Fe) best explained the fungal variation along the RDA axis 1 (Supplementary Fig. 1).

## Discussion

Peatlands contain a large diversity of microbial communities that differ in their resource use and the functional roles that they play in peatland ecosystems [65]. For example, fungi are critical in decomposing complex substrates [66], whereas bacteria are better at utilizing simple compounds [67]. While microbial biomass has been shown to be typically high across all peat depths, microbial composition varies with peat depth as it is shaped by many factors, including moisture and oxygen availability [68, 69], changes in substrate and redox potential [8, 70], and carbon and soil nutrient dynamics [65].

Our results demonstrated that prokaryote structure and function (i.e., predicted metabolic pathways) were significantly different between the acro-, meso-, and catotelm, consistent with our first hypothesis that there is a vertical stratification of prokaryote function in the intact bog soil. Prokaryote richness was highest in the intact bog mesotelm, which is in line with the previous studies that have proposed that the mesotelm forms a biogeochemical “hot spot” for microbial diversity and activity [15, 71]. We found that Proteobacteria and Acidobacteria were the dominant bacterial taxa across the acro-, meso-, and catotelm, which parallels previous studies that have suggested that Proteobacteria and Acidobacteria are primarily responsible for plant biomass degradation and nutrient cycling [72, 73]. Our study is one of the few ones to provide a comprehensive analysis of the microbial communities in Australian peatlands thus providing a baseline understanding for fungal and prokaryote assemblages in these peatlands.

Carbon (C), nitrogen (N), lead (Pb), sodium (Na), and manganese (Mn) collectively best explained the prokaryote variation as a function of soil depth in the intact bog. While we should be cautious to infer too much from correlative results in a small dataset, it is possible that the prokaryote structural and functional changes are related to the observed physico-chemical properties of peat that have been shown to change over peat depth profiles [74]. For example, we found high levels of manganese in the acrotelm. Manganese in natural environments is found as reduced soluble or adsorbed Mn (II) and insoluble Mn (III) and Mn (IV) oxides [75]. Many microorganisms, including bacteria and fungi, can oxidize Mn (II) or reduce Mn (III) or Mn (IV) oxides [76, 77]. Some authors have suggested that Mn oxides accumulated under oxic conditions, such as in the acrotelm, may be utilized by bacteria for respiration when conditions become anoxic [75, 78]. Based on our long-term water table level data, it is evident that in the intact bog, in the acrotelm conditions remain oxic most of the time, thus plausibly allowing for long-term Mn accumulation, whereas in the meso- and catotelm, where conditions become increasingly anoxic, the composition of bacterial communities may shift more towards bacteria that utilize Mn and many other inorganic and organic electron acceptors for respiration.

Prokaryote predicted metabolic pathways, similarly to their structure, were clustered by soil depth in the intact bog. This is in line with findings from other studies that have found functional differentiation of prokaryotes with soil depth in minerotrophic fens in China [8, 44]. Here, some of the most abundant predicted metabolic pathways in the meso- and catotelm were related to L-rhamnose degradation II, lipopolysaccharide biosynthesis pathway, sulfoquinovose degradation I, tetrahydromethanopterin (H_4_MPT) biosynthesis, and chorismate biosynthesis II (archaea). H_4_MPT is involved in methane production and it is produced by archaea [79, 80]. We uncovered two significant metabolic pathways that are related to methanogenesis, consistent with the fact that methanogens are common in environments where anaerobic biodegradation of organic material occurs, i.e., in deeper, more anoxic meso- and catotelms [81]. In the intact bog sites, the water table level was in the acrotelm for most of the growing season (i.e., November to March (Fig. 1d)), creating anoxic conditions in the meso- and catotelm where anaerobic biodegradation of organic material can occur.

The lower prokaryote richness observed in the dried peat acrotelm compared to the intact bog acrotelm is likely caused by the differences in the water table level. Additionally, it may be attributed to the differences in overall vegetation, but specifically *Sphagnum* moss cover. In the intact bog, we found that the *Sphagnum* cover ranged between 80 and 100%, whereas in the dried peat it was 30% or less. Our results are supported by findings from Wang et al. (2019), who also reported a correlation between vegetation (*Sphagnum* cover) and prokaryote community structure. Furthermore, we observed that the relative abundance of Chloroflexi was notably higher in the dried peat acrotelm. Bai et al. (2017) have reported that slow-growing Chloroflexi are more tolerant to depleted carbon resource availability which may be caused by changes in soil chemistry due to decomposition that burns off more labile carbohydrates [82]. It is also important to note that the differences in the acrotelm prokaryote communities may be due to the water table levels, as discussed previously. Specifically, the acrotelm in the dried peat is under the water most of the year creating anoxic conditions that may not be suitable to some prokaryotes.

In contrast to prokaryotes, soil fungal community structure was not affected by soil depth in the intact bog, consistent with Wang et al. (2019) who studied minerotrophic fens in China using a similar sampling design to ours, but in contrast with Lamit et al. (2017, 2021) who found dramatic shifts in fungal community structure with depth. Furthermore, it is important to note that marginal sampling effort in our study may have affected the detectability of an effect of soil depth on fungal communities, similarly to Wang et al. (2019) and in contrast to Lamit et al. (2017, 2021) where sampling effort was greater. This is farther reflected in the lack of overall compositional shifts in soil fungi in our study, although phyla and functional guilds changed with soil depth.

Here, we found that soil fungal structure was significantly explained by manganese (Mn), electrical conductivity (EC), and water table level (cm), which is supported by several other studies that have described the important link between nutrient resources, water table level, soil moisture, and soil fungi [83–86]. Specifically, soil fungi are highly susceptible to waterlogging and some fungal species are not able to tolerate anoxic conditions, which may result in considerably lower fungal richness in permanently waterlogged peatlands [87–89]. In our study site, water logging is unlikely to detrimentally affect the soil fungal communities in the bog peat acrotelm as the water table does not rise above the bog surface throughout the year, in contrast with the more variable water table in the dried peatland. We note that we did not assess physico-chemical properties of the intact or degraded soil, e.g., bulk density or microbial respiration, that would better inform soil activity. This clearly warrants further research.

Across our study sites, the dominant soil fungal phyla were Ascomycota, followed by Basidiomycota, which is in accordance with previous studies that have reported Ascomycota and Basidiomycota to dominate the soil fungal communities in peatlands globally, with average relative abundances of 46% and 40%, respectively [90]. According to our indicator species analysis, four soil fungal species associated significantly with the acro-, meso-, and catotelm in the intact bog. Specifically, *Trichoderma sp.* (Ascomycota), common soil fungi that are present in most soils, readily colonize plant roots and are known to exhibit mycoparasitism [91, 92], associated significantly with the acro- and mesotelm. *Hypochnicium huinayensis* (Basidiomycota) associated significantly with the meso- and catotelm and is a genus of corticoid, wood-inhabiting fungi with global distribution [93]. *Taphrina carpini* (Ascomycota) and *Symmetrospora gracilis* (Basidiomycota) associated significantly with the catotelm. Species from fungal genus *Taphrina* and *Symmetrospora* are yeast-like taxa, with *Taphrina sp*. known to cause plant diseases [94, 95]. Thus, fungal species from both Ascomycota and Basidiomycota associated significantly with all soil layers in the intact bog.

In the intact bog, plant pathogens and saprotrophs increased from ~ 25% in the acrotelm to ~ 95% in the catotelm. Saprotrophs are free-living filamentous fungi that decompose organic material and thus their dominance in the deepest and organic material rich soil layer, i.e., the catotelm, is not unexpected [18]. Saprotrophic fungi are the most abundant guild described in peatland studies [18, 90]. Earlier work on the carbon chemistry of both bog peat and the dried peat from Wellington Plains peatland by Grover and Baldock (2013) supports these findings. Grover and Baldock (2013) found that with increasing soil depth the proportion of o-alkyl carbon decreased, while the proportion of carbonyl carbon and aryl carbon increased. The alkyl:o-alkyl ratio, an index for the extent of decomposition [96], increased consistently with soil depth.

In the intact bog, the relative abundance of putative ectomycorrhizal, endophytic and orchid mycorrhizal fungi was highest in the mesotelm (~ 60%), followed by ~ 35% in the acrotelm. All fungi that classified as orchid mycorrhizal associations belonged to the genus *Serendipita* (family: Serendipitaceae) (Supplementary file 3), which are known to display highly diverse interactions with plants and include endophytes and other lineages that repeatedly evolved ericoid, orchid, and ectomycorrhizal abilities [97]. Several known plant species found in the intact bog form these associations (*A. alpina*, *E. paludosa*, *B. gunniana*, *E. minus* and *B. australe*, Supplementary Table 1). The most abundant vascular plants in the intact bog were rushes from the family Restionaceae, i.e., *Baloskion australe* (R. Br.) B. G. Briggs & L. A. S. Johnson (Mountain Cord-rush) and *Empodisma minus* (Hook. f.) L. A. S. Johnson & D. F. Cutler (Spreading rope-rush) (Table 1). Species from the family Restionaceae are known to be predominantly nonmycorrhizal in Australia, or forming arbuscular mycorrhizal associations occasionally (Supplementary Table 1) [98, 99].

We found that plant pathogens and saprotrophs were the most abundant guilds in the dried peat acrotelm (~ 70%), compared to ~ 30% in the intact bog. Saprotrophs play a central role in carbon dynamics as they decompose lignin and cellulose [100]. Differences in saprotroph abundance were attributed to variable vegetation cover and organic material content across intact bog and dried peat sites. Specifically, as mentioned above, in the intact bog, the *Sphagnum* cover ranged between 80 and 100%, whereas in the dried peat *Sphagnum* cover was 30% or less. Understory vascular plant species cover in the dried peat was overall similar to the intact bog plant cover, dominated by Restionaceae, with the addition of species from Poaceae family. Species from the Poaceae family are usually sparsely colonized by arbuscular mycorrhizal fungi and some species are also nonmycorrhizal (Supplementary Table 1) [101]. Furthermore, the dried site cores had a much lower abundance of the ectomycorrhizal Myrtaceae genus *Baeckea*, likely explaining the decline in guilds that contain ectomycorrhizal fungi in the dried site cores relative to the intact site cores.

## Conclusions

The microbial communities in Australian peatlands are extremely understudied and we lack a baseline knowledge on the microbial diversity in these soils. However, it is important to better understand the soil microbial diversity in peatlands, the effect of degradation on soil microbial composition, and responses to environmental changes as it impacts the C storage ability of these peatlands. Our study has demonstrated that both fungal and prokaryote communities were significantly shaped by soil abiotic factors. Peatland degradation reduced prokaryote richness and shifted the relative abundance of fungal guilds towards more saprotrophic communities. Thus, current and future changes to vegetation and environmental conditions in these peatlands are likely to lead to altered microbial structure and associated functions, which may in turn have implications for broader ecosystem function changes in these peatlands.

## Supplementary Information

Below is the link to the electronic supplementary material.

Supplementary file1 (DOCX 93 KB)

Supplementary file2 (XLSX 54 KB)

Supplementary file3 (XLSX 75 KB)

Supplementary file4 (XLSX 524 KB)

## Data Availability

Data is available in the Supplementary files and code is available upon request.
