# Supplementary material for: Degradation Reduces Microbial Richness and Alters Microbial Functions in an Australian Peatland
Source: Microb Ecol. 2022 Jul 22;85(3):875–91. doi: 10.1007/s00248-022-02071-z (PMC10156627; doi:10.1007/s00248-022-02071-z)
Supplement: Supplementary file 1 — Supplementary file1 (DOCX 93 KB) [file 248_2022_2071_MOESM1_ESM.docx]

**Supplementary material**

**Supplementary Table 1.** Mycorrhizal associations of dominant plants found in the studied intact and dried peat sites at Wellington Plains peatland, Australia.

| **Species** | **Family** | **Form** | **Occurrence** | **Mycorrhizal status** | **Reference** |
| --- | --- | --- | --- | --- | --- |
| *Astelia alpina* | Asteliaceae | Herb | Intact | Arbuscular mycorrhizal | Johnson (1977) |
| *Epacris paludosa* | Epacridaceae | Shrub | Intact | Ericoid | Cairney & Ashford (2002); Bellgard (1991) |
|  |  |  |  |  |  |
| *Baeckea gunniana* | Myrtaceae | Shrub/tree | Intact/Dried | Ecto mycorrhizal | Brundrett et al. (1995), McGee (1986), Bellgard (1991) |
| *Empodisma minus* | Restionaceae | Rush | Intact/Dried | Nonmycorrhizal/arbuscular mycorrhizal | Meney et al (1993); Brundrett and Tedersoo (2018) |
| *Baloskion australe* | Restionaceae | Rush | Intact/Dried | Nonmycorrhizal/arbuscular mycorrhizal | Meney et al (1993); Brundrett and Tedersoo (2018) |
|  |  |  |  |  |  |
| *Hydrocotyle sp.* | Araliaceae | Herb | Dried | Arbuscular mycorrhizal | Brundrett & Abbott (1991) |
| *Solenogyne dominii* | Asteraceae | Herb | Dried | Arbuscular mycorrhizal | Brundrett & Abbott (1991) |
| *Celmisia costiniana* | Asteraceae | Herb | Dried | Arbuscular mycorrhizal | Brundrett & Abbott (1991) |
| *Epacris celata* | Epacridaceae | Shrub | Dried | Ericoid | Cairney & Ashford (2002); Bellgard (1991) |
| *Baeckea utilis* | Myrtaceae | Shrub | Dried | Arbuscular mycorrhizal | Brundrett et al. (1995) |
| *Poa sp.* | Poaceae | Grass | Dried | Nonmycorrhizal/arbuscular mycorrhizal | Brundrett (2008) |
| *Viola betonicifolia* | Violaceae | Herb | Dried | Arbuscular mycorrhizal | McGee (1986), Brundrett et al. (1995) |

References: Johnson PN. 1977. Mycorrhizal Endogonaceae in a New Zealand forest. *New Phytologist***78:**161-170. Cairney JWG, Ashford AE. 2002. Biology of mycorrhizal associations of epacrids (Ericaceae). *New Phytologist***154:** 305-326. Bellgard SE. 1991. Mycorrhizal associations of plant species in Hawksbury sandstone vegetation. *Australian Journal of Botany* **39:** 357-364. Brundrett M, Abbott L, Jasper D, Malajczuk N, Bougher N, Brennan K, Ashwath N. 1995. *Mycorrhizal Associations in the Alligator Rivers Region. Part II Results of Experiments.* Final Report. Office of the Supervising Scientist, Jabiru N. T.. Meney KA, Dixon KW, Scheltema M, Pate JS. 1993. Occurrence of vesicular mycorrhizal fungi in dryland species of Restionaceae and Cyperaceae from south-west Western Australia. *Australian Journal of Botany* **41**: 733-737. Brundrett, M. C., & Tedersoo, L. (2018). Evolutionary history of mycorrhizal symbioses and global host plant diversity. *New Phytologist*, *220*(4), 1108-1115. Brundrett MC, Abbott LK. 1991. Roots of jarrah forest plants. I. Mycorrhizal associations of shrubs and herbaceous plants. *Australian Journal of Botany* **39:** 445-457. Brundrett, M. (2008). Mycorrhizal associations: the web resource (<http://www.mycorrhizas.info>). McGee PA. 1986. Mycorrhizal associations of plant species in a semiarid community. *Australian Journal of Botany***34**: 585-593.

**Supplementary file2.** Full list of 420 PiCRUST predicted metabolic pathways for prokaryotes at Wellington Plains peatland, Australia.

**Supplementary file3.** Fungal taxonomic assignments, including FUNguild output for fungal guilds from intact bog and dried peat soils at Wellington Plains peatland, Australia.

**Supplementary file4.** Full list of dominant prokaryote phyla and their relative abundances from intact bog and dried peat soils at Wellington Plains peatland, Australia.

**Supplementary Table 5.** Summary of redundancy analysis (dbRDA) for the effects of environmental variables on fungal composition in intact bog soil from the acrotelm, mesotelm and catotelm based on extracted DNA from soil from Wellington Plains peatland, Australia. Summary statistics for five redundancy axes are presented. PVE = percentage constrained variation explained.

| **RDA axis** | **Eigenvalue** | **PVE** | ***F*** | **Df num, den** | | ***P*** |
| --- | --- | --- | --- | --- | --- | --- |
| Axis 1 | 0.026 | 7.68 | 2.15 | | 1,4 | 0.001 |
| Axis 2 | 0.021 | 6.06 | 1.70 | | 1,4 | 0.057 |
| Axis 3 | 0.015 | 4.51 | 1.26 | | 1,4 | 0.533 |
| Axis 4 | 0.010 | 4.33 | 1.22 | | 1,4 | 0.364 |

**Supplementary Table 6.** Summary of permutation test for dbRDA under reduced model with 999 permutations for five soil chemistry and hydrology variables tested in the fungal dbRDA analysis.

| **Variable** | **Df** | **Variance** | ***F*** | ***P*** |
| --- | --- | --- | --- | --- |
| Manganese (Mn) | 1 | 0.018 | 1.473 | 0.024 |
| Electrical conductivity (EC) | 1 | 0.017 | 1.408 | 0.038 |
| Water table level (cm) | 1 | 0.017 | 1.407 | 0.038 |
| Nitrogen (N) | 1 | 0.015 | 1.241 | 0.133 |
| Residual | 4 |  |  |  |

**Supplementary Table 7.** Summary of redundancy analysis (dbRDA) for the effects of soil chemistry and hydrology variables on prokaryote composition in intact bog soil from the acrotelm, mesotelm and catotelm based on extracted DNA from soil from Wellington Plains peatland, Australia. Summary statistics for five redundancy axes are presented. PVE = percentage constrained variation explained.

| **RDA axis** | **Eigenvalue** | **PVE** | ***F*** | **Df num, den** | | ***P*** |
| --- | --- | --- | --- | --- | --- | --- |
| Axis 1 | 0.035 | 8.85 | 2.80 | | 1,4 | 0.009 |
| Axis 2 | 0.021 | 5.34 | 1.69 | | 1,4 | 0.221 |
| Axis 3 | 0.014 | 3.70 | 1.17 | | 1,4 | 0.852 |
| Axis 4 | 0.011 | 2.93 | 0.92 | | 1,4 | 0.875 |
| Axis 5 | 0.010 | 2.75 | 0.87 | | 1,4 | 0.564 |

**Supplementary Table 8.** Summary of permutation test for dbRDA under reduced model with 999 permutations for five soil chemistry and hydrology variables tested in the prokaryote dbRDA analysis.

| **Variable** | **Df** | **Variance** | ***F*** | ***P*** |
| --- | --- | --- | --- | --- |
| Nitrogen (N) | 1 | 0.013 | 1.013 | 0.430 |
| Manganese (Mn) | 1 | 0.012 | 0.975 | 0.463 |
| Carbon (C) | 1 | 0.011 | 0.941 | 0.536 |
| Sodium (Na) | 1 | 0.014 | 1.106 | 0.324 |
| Lead (Pb) | 1 | 0.013 | 1.018 | 0.427 |
| Residual | 3 | 0.037 |  |  |

**Supplementary Figure 1.** dbRDA biplot for fungal communities in intact bog (n = 3) and dried peat (n = 2) soil from the acrotelm (10 cm) based on extracted DNA from soil from Wellington Plains peatland, Australia.

**Supplementary Figure 2.** Mean and ± S.E for Manganese (Mn), mg/kg, Carbon (C) %, Nitrogen (N) %, Sodium Na (%) and Lead (Pb) mg/kg from intact bog acrotelm (10 cm), mesotelm (30 cm) and catotelm (60cm) soil samples collected from Wellington Plains peatland, Australia.
